# Pediatric pulmonary arterial hypertension: global epidemiology and disease burden during the period 1990 to 2021

**DOI:** 10.3389/fcvm.2025.1544545

**Published:** 2025-08-29

**Authors:** Ying Chen, Rongguo Zhang, Yongxiao Zheng, Chenghong Li, Xiaobei Wang, Zhongmei Wen, Fajiu Li

**Affiliations:** ^1^ Department of Pulmonary and Critical Care Medicine, The Sixth Hospital of Wuhan, Affiliated Hospital of Jianghan University, Wuhan, Hubei, China; ^2^ Department of Biostatistics, School of Public Health, Cheeloo College of Medicine, Shandong University, Jinan, Shandong, China; ^3^ Institute for Medical Dataology, Cheeloo College of Medicine, Shandong University, Jinan, Shandong, China; ^4^ Department of Respiratory Medicine, The First Hospital of Jilin University, Changchun, Jilin, China

**Keywords:** pulmonary arterial hypertension, epidemiology, pediatric, global, disease burden

## Abstract

**Objectives:**

Pediatric pulmonary arterial hypertension (PAH) is a severe and potentially fatal disease; however, the global epidemiological burden of pediatric PAH has not been comprehensively elucidated.

**Study design:**

The Global Burden of Diseases Study 2021 has incorporated PAH for the first time. This study analyzed the incidence, prevalence, mortality, and disability-adjusted life years (DALYs) associated with pediatric PAH from 1990 to 2021.

**Results:**

Globally, the age-standardized prevalence rate (ASPR) of pediatric PAH in children aged 0–14 years showed a relatively stable, from 0.44 per 100,000 in 1990 to 0.43 per 100,000 in 2021. Females exhibited higher age-standardized incidence rates (ASIR) and ASPR compared to males. The ASIR of pediatric PAH increased modestly, with an average annual growth of 0.12%. Children aged 10–14 years had the highest prevalence, with rates of 0.66 per 100,000 in 1990 and 0.65 per 100,000 in 2021. In contrast, newborns aged 0–6 days experienced the highest mortality rates (34.16 and 13.67 per 100,000) and DALYs rates (3,073.56 and 1,229.88 per 100,000) during the same period. Countries with high-middle SDI levels had the highest ASPR, while high-SDI countries reported the lowest age-standardized mortality rate and age-standardized disability rate.

**Conclusions:**

Globally, prevalence, mortality, and DALYs of pediatric PAH show a declining trend from 1990 to 2021. Females exhibited higher incidence and prevalence rate but lower mortality and DALYs. 10–14 years had the highest prevalence, whereas high PAH-related mortality in neonates remains a critical concern. Tailored health policies for PAH management are needed.

## Introduction

Pulmonary arterial hypertension (PAH) is a multifactorial pathophysiological condition associated with a spectrum of cardiovascular and respiratory diseases. It can manifest at any age, including in pediatric populations (1). Previous guidelines have defined pediatric PAH as a mean pulmonary artery pressure of ≥25 mmHg at rest, measured by right heart catheterization in children older than three months at sea level, with a pulmonary capillary wedge pressure ≤15 mmHg and a pulmonary vascular resistance index ≥3 Wood units·m^2^ (2). In 2018, the World Symposium on Pulmonary Hypertension revised the diagnostic criteria for PAH to a mean pulmonary artery pressure >20 mmHg (3). The pediatric task force recommends the adoption of the same diagnostic criteria for children (4). In recent decades, the understanding of pediatric PAH has significantly advanced, encompassing its pathobiology, molecular mechanisms, and therapeutic approaches (5–7). However, epidemiological data on pediatric PAH were relatively sparse, primarily sourced from developed European and North American nations at a national level (8–16). The estimated incidence varies from 1.6 to 3.0 per 1,000,000 children, while the prevalence ranges from 9.8 to 20 per 1,000,000 children (11, 17). A study from Turkey, a developing country, reported the incidence of PAH in Turkish children. The overall incidence of idiopathic pulmonary hypertension in childhood was 11.7 cases per million, and secondary pulmonary hypertension was 9.5 cases per million (14), which is significantly higher than in Western countries. Additionally, there is limited data on the overall incidence of PAH, as most studies focus on specific subtypes of the disease.

The Global Burden of Disease (GBD) study is a collaborative international effort designed to generate consistent and comparable metrics for assessing disease burden across national and subnational populations (18). The GBD 2021 study provides the first-ever estimates of the incidence, prevalence, mortality, and disability-adjusted life years (DALYs) associated with PAH, analyzed at global, regional, and national levels from 1990 to 2021, with stratification by sociodemographic status, age, and sex. This study aims to investigate the global burden of pediatric PAH, providing critical insights to guide effective interventions and optimize resource allocation to mitigate the impact of this debilitating condition.

## Methods

### Study population and data collection

In the GBD 2021 study, we conducted an analysis of repeated cross-sectional data obtained from the most recent Global Health Data Exchange (http://ghdx.healthdata.org/gbd-results-tool) covering the period from 1990 to 2021. This dataset includes newly incorporated diseases, such as PAH (ICD-9 416.0 or ICD-10 I27.0), across 204 countries and territories, as well as 811 subnational locations. We extracted data on pediatric PAH (0–14 years), including location-, age-, and sex-specific incidence, prevalence, mortality, and DALYs, along with corresponding 95% uncertainty intervals (UIs). To account for comorbidities, we employed micro-simulation techniques to derive the final estimate of years lived with disability. DALYs were calculated as the sum of years lived with disability and years of life lost. The detailed methodology utilized in GBD 2021 has been described in previous publications (19).

We also calculated the sociodemographic index (SDI) for each country to assess the social and economic factors influencing health outcomes. The SDI ranges from 0 to 1, where a value of 1 indicates the highest levels of education, highest per capita income, and lowest fertility rates. In our analysis, SDI values were categorized into five groups: low, low-middle, middle, high-middle, and high.

### Statistical analysis

The GBD 2021 study integrated data from systematic reviews of published literature, survey data, and longitudinal studies into the Bayesian meta-regression tool (DisMod-MR 2.1) to estimate various indicators of disease burden for pediatric PAH. To account for demographic variations across different regions and time periods, age-standardized incidence rates (ASIR), prevalence rates (ASPR), mortality rates (ASMR), and DALY rates (ASDR) were calculated using the 2017 World Standard Population, with corresponding 95% uncertainty intervals (UIs). These rates were standardized per 100,000 individuals, and the 95% UI was derived from the 2.5th and 97.5th percentiles of 1,000 samples drawn from the posterior distributions of each metric.

To evaluate the temporal trends in the incidence, prevalence, mortality, and DALY rates of pediatric PAH, average annual percent change (AAPC) and the corresponding 95% confidence intervals (CI) were calculated using the Joinpoint Regression Program software (Version 4.9.0.0, National Cancer Institute, USA) (20). A Monte Carlo permutation method was employed for significance testing, and the AAPC was determined by weighting the annual percent change of each segment in the final significant model (21). All other analyses were conducted using R software (Version 4.4.3). A *P-*value of less than 0.05 was considered statistically significant.

## Results

### Global trends

**Figure 1:**
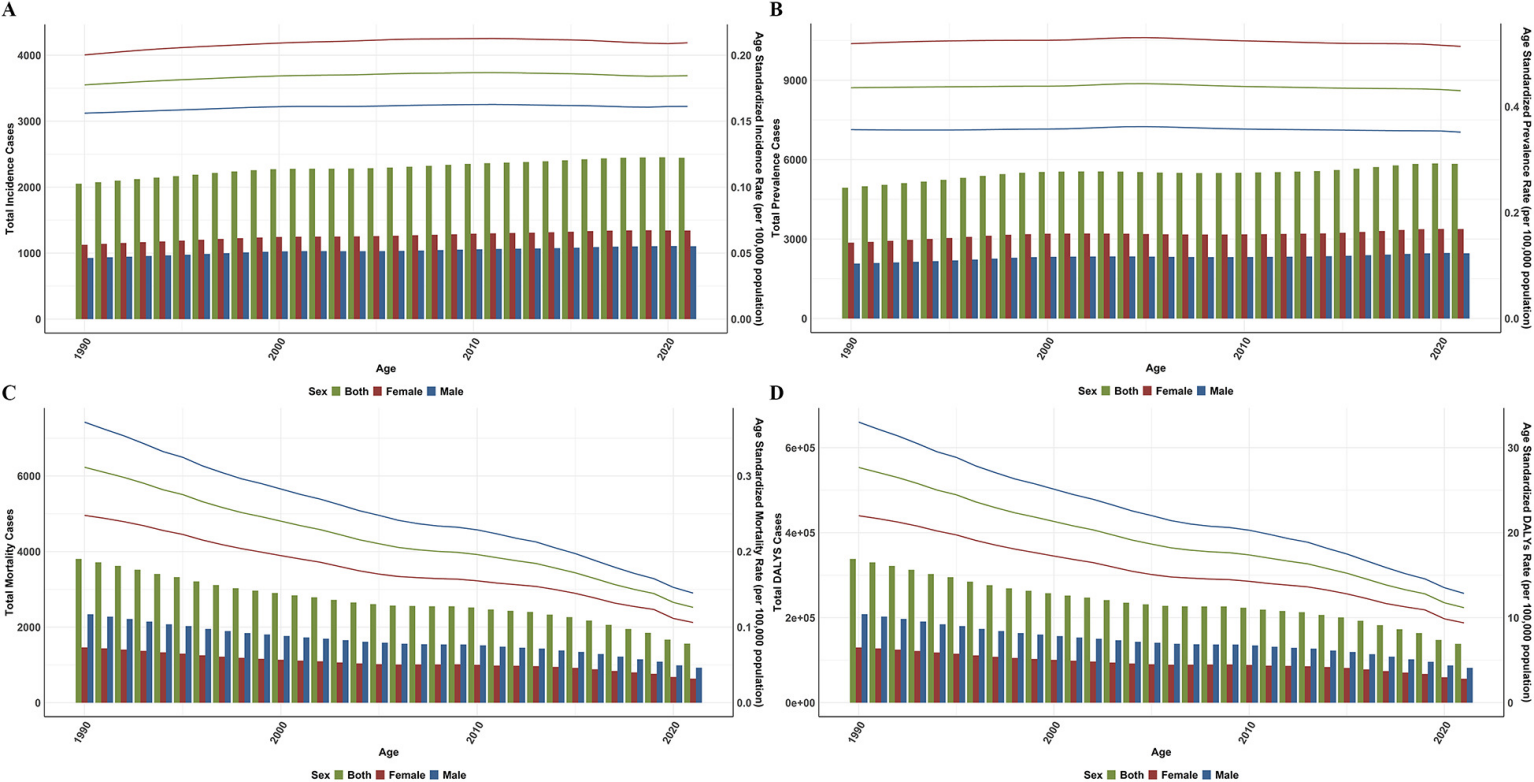
Global cases and age-standardized rates of pulmonary artery hypertension incidence **(A)**, prevalence **(B)**, mortality **(C)**, and disability-adjusted life years **(D)** by sex from 1990 to 2021.

Globally, the incidence of pediatric PAH in children aged 0–14 years increased by 3.93% between 1990 and 2021, rising from 2,051.52 to 2,445.57 cases. The ASIR of pediatric PAH demonstrated a modest increase, from 0.18 [95% uncertainty interval (UI): 0.17, 0.19] per 100,000 population in 1990–0.18 (95% UI: 0.18, 0.19) per 100,000 in 2021, with an average annual increase of 0.12% [95% confidence interval (CI): 0.12, 0.13]. In contrast to the ASIR (Figure 1A), the ASPR (Figure 1B), ASMR (Figure 1C), and ASDR (Figure 1C) exhibited declining trends, with AAPC of −0.04% (95% CI: −0.04%, −0.04%), −2.91% (95% CI: −2.95%, −2.88%), and −2.92% (95% CI: −2.96%, −2.89%) between 1990 and 2021, respectively. The ASPR of pediatric PAH was 0.44 (95% UI: 0.42, 0.45) per 100,000 population in 1990, relatively stable in 2021 with 0.43 (95% UI: 0.42, 0.44) per 100,000. Correspondingly, the ASMR dropped from 0.31 (95% UI: 0.30, 0.32) to 0.13 (95% UI: 0.12, 0.13) per 100,000 population. The decline in ASMR was significantly greater than that of the ASPR, showing a reduction approximately 72.75 times greater. ASDR decreased by 59.61%, from 27.68 per 100,000 population in 1990 to 11.18 per 100,000 population in 2021 (Table 1).

**Table 1:** Age-standardized prevalence and AAPC of PAH in people aged 0-14 years at global and regional level, 1990-2021.

	Incidence (95%UI)				Percent change（%）	AAPC % (95% CI)	*P*
	No of pediatric PAH in 1990	Age standardized rate in 1990 (per 100,000)	No of pediatric PAH in 2021	Age standardized rate in 2021 (per 100,000)			
Global	2,051.52	0.18 (0.17, 0.19)	2,445.57	0.18 (0.18, 0.19)	3.93	0.12 (0.12, 0.13)	<0.001
Gender							
Male	926.11	0.16 (0.15, 0.17)	1,103.15	0.16 (0.15, 0.17)	3.26	0.11 (0.1, 0.11)	<0.001
Female	1,125.41	0.2 (0.19, 0.21)	1,342.42	0.21 (0.2, 0.22)	4.59	0.14 (0.14, 0.14)	<0.001
SDI level							
High SDI	148.29	0.12 (0.1, 0.14)	136.13	0.12 (0.1, 0.14)	−0.52	−0.02 (−0.02, −0.01)	<0.001
High-middle SDI	262.00	0.14 (0.13, 0.16)	217.92	0.15 (0.13, 0.17)	2.58	0.09 (0.07, 0.09)	<0.001
Middle SDI	642.81	0.17 (0.15, 0.18)	626.61	0.17 (0.16, 0.18)	1.16	0.04 (0.03, 0.04)	<0.001
Low-middle SDI	595.46	0.19 (0.18, 0.21)	738.00	0.19 (0.18, 0.21)	0.13	0 (0, 0.01)	0.50
Low SDI	401.37	0.26 (0.24, 0.29)	725.11	0.24 (0.22, 0.25)	−10.52	−0.36 (−0.37, −0.35)	<0.001
	Prevalence (95%UI)						
Global	4,939.62	0.44 (0.42, 0.45)	5,840.35	0.43 (0.42, 0.44)	−1.24	−0.04 (−0.04, −0.04)	<0.001
Gender							
Male	2,075.32	0.36 (0.34, 0.37)	2,463.57	0.35 (0.34, 0.37)	−1.34	−0.04 (−0.04, −0.03)	<0.001
Female	2,864.30	0.52 (0.5, 0.54)	3,376.78	0.51 (0.5, 0.53)	−0.97	−0.03 (−0.04, −0.03)	<0.001
SDI level							
High SDI	664.07	0.53 (0.49, 0.57)	624.71	0.52 (0.48, 0.56)	−1.5	−0.05 (−0.05, −0.04)	<0.001
High-middle SDI	1,005.31	0.54 (0.51, 0.58)	873.47	0.56 (0.52, 0.59)	2.09	0.06 (0.05, 0.07)	<0.001
Middle SDI	1,674.81	0.44 (0.42, 0.46)	1,794.26	0.46 (0.44, 0.49)	5.88	0.19 (0.18, 0.19)	<0.001
Low-middle SDI	1,049.44	0.35 (0.33, 0.37)	1,466.97	0.37 (0.35, 0.39)	6.98	0.22 (0.22, 0.23)	<0.001
Low SDI	540.50	0.38 (0.35, 0.42)	1,076.10	0.36 (0.34, 0.38)	−6.31	−0.22 (−0.23, −0.21)	<0.001
	Mortality(95%UI)						
Global	3,803.85	0.31 (0.3, 0.32)	1,563.36	0.13 (0.12, 0.13)	−59.48	−2.91 (−2.95, −2.88)	<0.001
Gender							
Male	2,340.43	0.37 (0.36, 0.39)	925.43	0.15 (0.14, 0.15)	−60.92	−3.02 (−3.05, −2.99)	<0.001
Female	1,463.42	0.25 (0.24, 0.26)	637.93	0.11 (0.1, 0.11)	−57.19	−2.74 (−2.79, −2.7)	<0.001
SDI level							
High SDI	191.90	0.16 (0.14, 0.19)	71.68	0.07 (0.05, 0.09)	−56.46	−2.67 (−2.81, −2.56)	<0.001
High-middle SDI	537.97	0.31 (0.28, 0.34)	88.78	0.07 (0.06, 0.09)	−76.28	−4.56 (−4.59, −4.53)	<0.001
Middle SDI	1,040.71	0.27 (0.25, 0.29)	248.41	0.08 (0.07, 0.09)	−70.97	−3.93 (−3.99, −3.88)	<0.001
Low-middle SDI	1,510.42	0.43 (0.41, 0.46)	628.15	0.17 (0.16, 0.18)	−60.52	−3.01 (−3.04, −2.97)	<0.001
Low SDI	519.64	0.28 (0.25, 0.30)	524.37	0.16 (0.15, 0.18)	−41.87	−1.72 (−1.75, −1.7)	<0.001
	DALYs (95%UI)						
Global	338,152.80	27.68 (27.59, 27.77)	138,285.34	11.18 (11.12, 11.24)	−59.61	−2.92 (−2.96, −2.89)	<0.001
Gender							
Male	208,220.31	33.01 (32.87, 33.15)	81,943.84	12.86 (12.77, 12.95)	−61.04	−3.02 (−3.06, −3.00)	<0.001
Female	129,932.49	22 (21.88, 22.12)	56,341.50	9.39 (9.31, 9.47)	−57.32	−2.75 (−2.79, −2.71)	<0.001
SDI level							
High SDI	16,957.11	14.24 (14.02, 14.45)	6,319.33	6.2 (6.05, 6.35)	−56.46	−2.67 (−2.81, −2.55)	<0.001
High-middle SDI	47,731.21	27.35 (27.10, 27.59)	7,856.60	6.49 (6.34, 6.63)	−76.27	−4.56 (−4.62, −4.52)	<0.001
Middle SDI	92,436.83	23.84 (23.69, 23.99)	21,967.75	6.91 (6.82, 7.00)	−71.01	−3.93 (−4.00, −3.88)	<0.001
Low-middle SDI	134,652.30	38.52 (38.31, 38.73)	55,693.59	15.15 (15.03, 15.28)	−60.66	−3.02 (−3.05, −2.99)	<0.001
Low SDI	46,089.23	24.51 (24.28, 24.73)	46,272.69	14.2 (14.07, 14.33)	−42.05	−1.73 (−1.76, −1.71)	<0.001

No, number; AAPC, average annual percentage change; CI, confidence interval; SDI, sociodemographic index; PAH=, pulmonary arterial hypertension; UI, uncertainty interval.

### Global trends by sex

In 1990, the global incidence of pediatric PAH in males was 1.22 times higher than in females, with 926.11 and 1,125.41 cases, respectively. By 2021, the incidence had increased in both genders, reaching 1,103.15 cases in males and 1,342.42 cases in females. In terms of the ASIR, in 1990, males had an ASIR of 0.16 (95% UI: 0.15, 0.17) per 100,000, and females had 0.20 (95% UI: 0.19, 0.21) per 100,000. By 2021, the ASIR for males remained at 0.16 (95% UI: 0.15, 0.17) per 100,000, while for females, it increased to 0.21 (95% UI: 0.20, 0.22) per 100,000. The AAPC was 0.11% (95% CI: 0.10, 0.11) for males and 0.14% (95% CI: 0.14, 0.14) for females (Figure 1A). From 1990 to 2021, the ASPR of PAH in both male and female children showed a declining trend, with an AAPC of −1.34% for males and −0.97% for females. In 1990, the ASPR for males was 0.36 (95% UI: 0.34, 0.37) per 100,000, and for females, it was 0.52 (95% UI: 0.50, 0.54) per 100,000. By 2021, these ASPR had slightly decreased to 0.35 (95% UI: 0.34, 0.37) per 100,000 for males and 0.51 (95% UI: 0.50, 0.53) per 100,000 for females (Figure 1B). From 1990 to 2021, the ASMR for males decreased from 0.37 (0.36, 0.39) per 100,000 to 0.15 (0.14, 0.15) per 100,000, while for females, it declined from 0.25 (0.24, 0.26) per 100,000 to 0.11 (0.10, 0.11) per 100,000. The AAPC was −3.02% (95% CI: −3.05, −2.99) for males and −2.74% (95% CI: −2.79, −2.70) for females (Figure 1C). Regarding DALYs, both males and females experienced a significant reduction between 1990 and 2021. The ASDR for males decreased from 33.01 (32.87, 33.15) per 100,000 in 1990 to 12.86 (12.77, 12.95) per 100,000 in 2021, with an AAPC of −3.02%. For females, the ASDR declined from 22.00 (21.88, 22.12) per 100,000 to 9.39 (9.31, 9.47) per 100,000, with an AAPC of −2.75% (Table 1, Figure 1D).

### Global trends by age

Between 1990 and 2021, the prevalence rate of pediatric PAH in the 0–14 years exhibited a steady upward trend. The highest prevalence was observed in the 10–14 years subgroup, with rates of 0.66 (95% UI: 0.93, 0.44) per 100,000 population in 1990 and 0.65 (95% UI: 0.92, 0.43) per 100,000 in 2021. The estimated number of cases was 3,535.88 (95% UI: 4,992.66, 2,364.52) in 1990 and 4,317.65 (95% UI: 6,154.21, 2,876.12) in 2021. In contrast to the rising prevalence, the mortality rate of pediatric PAH declines with age. Newborns aged 0–6 days experience the highest mortality, with rates of 34.16 (95% UI: 43.07, 25.73) per 100,000 in 1990, dropping significantly to 13.67 (95% UI: 18.55, 10.28) per 10,000 by 2021. Despite decline, mortality rates in this subgroup remain the highest. In contrast, the mortality rate in the 10–14 years subgroup was the lowest, at 0.04 (95% UI: 0.04, 0.03) per 100,000 in 1990 and 0.02 (95% UI: 0.03, 0.02) per 100,000 in 2021. Similar to the trend observed in mortality, newborns aged 0–6 days had the highest DALYs rate, recorded at 3,073.56 (95% UI: 3,875.46, 2,315.32) per 100,000 in 1990 and 1,229.88 (95% UI: 1,668.78, 925.17) per 100,000 in 2021(Supplementary Table S1).

### Global trends by sociodemographic index

**Figure 2:**
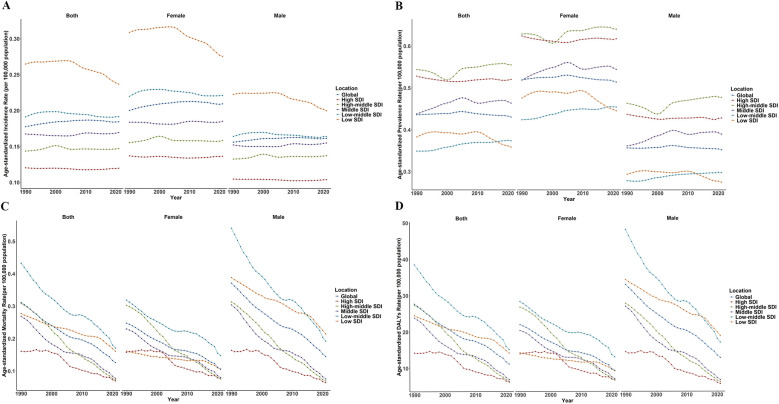
Temporal trend of age-standardized rates of incidence **(A)**, prevalence **(B)**, mortality **(C)**, and disability-adjusted life years **(D)** of pulmonary artery hypertension from 1990 to 2021 at global socio-demographic index (SDI) levels.

Globally, countries or territories classified as Low-SDI exhibited the highest ASIR of pediatric PAH, while those categorized as High-SDI demonstrate the lowest ASIR. In 1990, the ASIR was 401.37 per 100,000 population in Low-SDI regions, compared to 148.29 per 100,000 in High-SDI countries. By 2021, these figures had declined to 0.24 and 0.12 per 100,000 population, respectively. Notably, from 1990 to 2021, only Low-SDI and High-SDI countries experienced negative AAPC of −0.36% and −0.02%, respectively, while all other subgroups faced positive growth trends (Table 1; Figure 2A). Regarding ASPR, trends from 1990 to 2021 indicate that only Low-SDI and High-SDI countries demonstrated reduction with AAPCs of −0.22% and −0.05%, respectively. High-middle SDI regions reported the highest ASPR, with values of 0.54 (95% UI: 0.51, 0.58) per 100,000 in 1990 and 0.56 (95% UI: 0.52, 0.59) per 100,000 in 2021. In 2021, Low-SDI regions had the lowest prevalence among the five subgroups (Table 1; Figure 2B). Over the past 32 years, both the ASMR and ASDR for pediatric PAH have shown a consistent annual decline. Low-middle SDI regions recorded the highest ASMR, at 0.43 (95% UI: 0.41, 0.46) per 100,000 in 1990 and 0.17 (95% UI: 0.16, 0.18) per 100,000 in 2021, while High-SDI regions had the lowest ASMR, with values of 0.16 (95% UI: 0.14, 0.19) and 0.07 (95% UI: 0.05, 0.09) per 100,000, respectively. High-middle SDI regions demonstrated the fastest decline in ASMR, with an AAPC of −4.56%, whereas Low-SDI regions exhibited the slowest decline, with an AAPC of −1.72% (Table 1; Figure 2C). Similarly, Low-middle SDI regions reported the highest ASDR, with values of 38.52 (95% UI: 38.31, 38.73) in 1990 and 15.15 (95% UI: 15.03, 15.28) in 2021, resulting in an AAPC of −3.02 (95% UI: −3.05, −2.99). High-middle SDI regions also experienced a rapid decline of −4.56% (95% UI: −4.62, −4.52) (Table 1, Figure 2D).

### Regional and national trends

**Figure 3:**
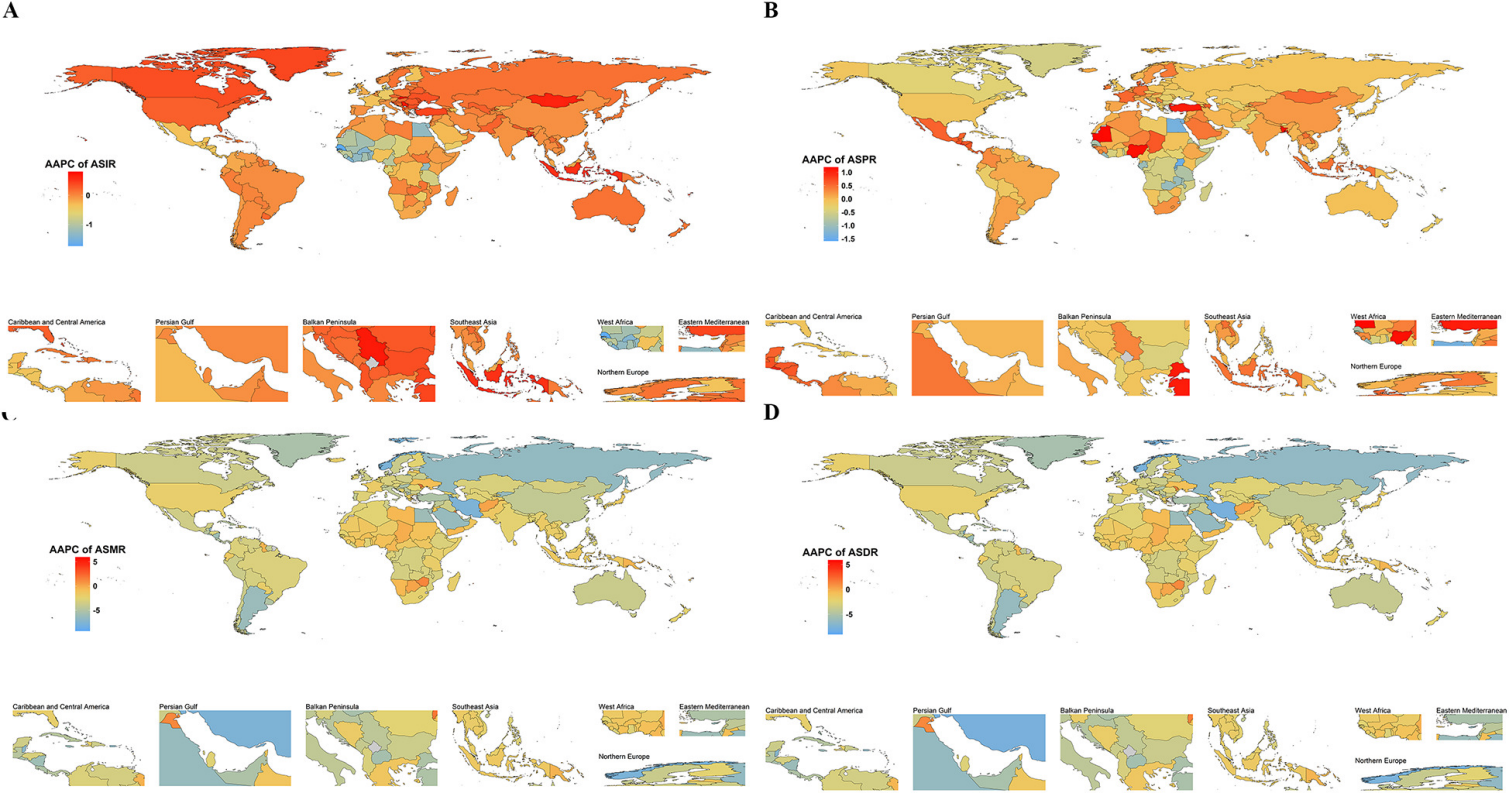
Map showing average annual percentage change (AAPC) in global incidence **(A)**, prevalence **(B)**, mortality **(C)**, and disability-adjusted life years **(D)** of pulmonary artery hypertension, 1990-2021. World map from: http://www.resdc.cn/.

Among the global 21 regions, 10 exhibited a declining trend in ASIR of pediatric PAH, while 11 regions showed an increasing trend. Eastern Sub-Saharan Africa had the highest ASIR, recorded at 0.32 (95% UI: 0.28, 0.37) per 100,000 population in 1990 and 0.29 (95% UI: 0.26, 0.32) per 100,000 in 2021. In contrast, High-Income North America reported the lowest ASIR, with values of 0.09 (95% UI: 0.06, 0.12) per 100,000 in 1990 and 0.09 (95% UI: 0.07, 0.13) per 100,000 in 2021. Eastern Europe had the highest ASPR of pediatric PAH globally, recorded at 0.79 (95% UI: 0.69, 0.89) per 100,000 in 1990 and 0.76 (95% UI: 0.66, 0.88) per 100,000 in 2021. Conversely, South Asia reported the lowest ASPR, with values of 0.31 (95% UI: 0.29, 0.33) per 100,000 in 1990 and 0.33 (95% UI: 0.31, 0.34) per 100,000 in 2021. Globally, all regions exhibit a consistent annual decline in both ASMR and ASDR for pediatric PAH. North Africa and the Middle East had the highest ASMR of pediatric PAH globally, while Central Europe has the lowest ASMR. In 1990, the ASMR for North Africa and the Middle East was 1.23 (95% UI: 1.16, 1.30) per 100,000, which was 24.60 times higher than that of Central Europe. By 2021, this ratio had decreased to 16.00 times. The largest reduction in DALYs from pediatric PAH among 0–14years was in Eastern Europe (AAPC −5.74%). In 2019, the highest ASDR was in North Africa and Middle East (10.49 per 100,000) and the lowest was in Central Europe (4.11 per 100,000) (Figure 3).

**Figure 4:**
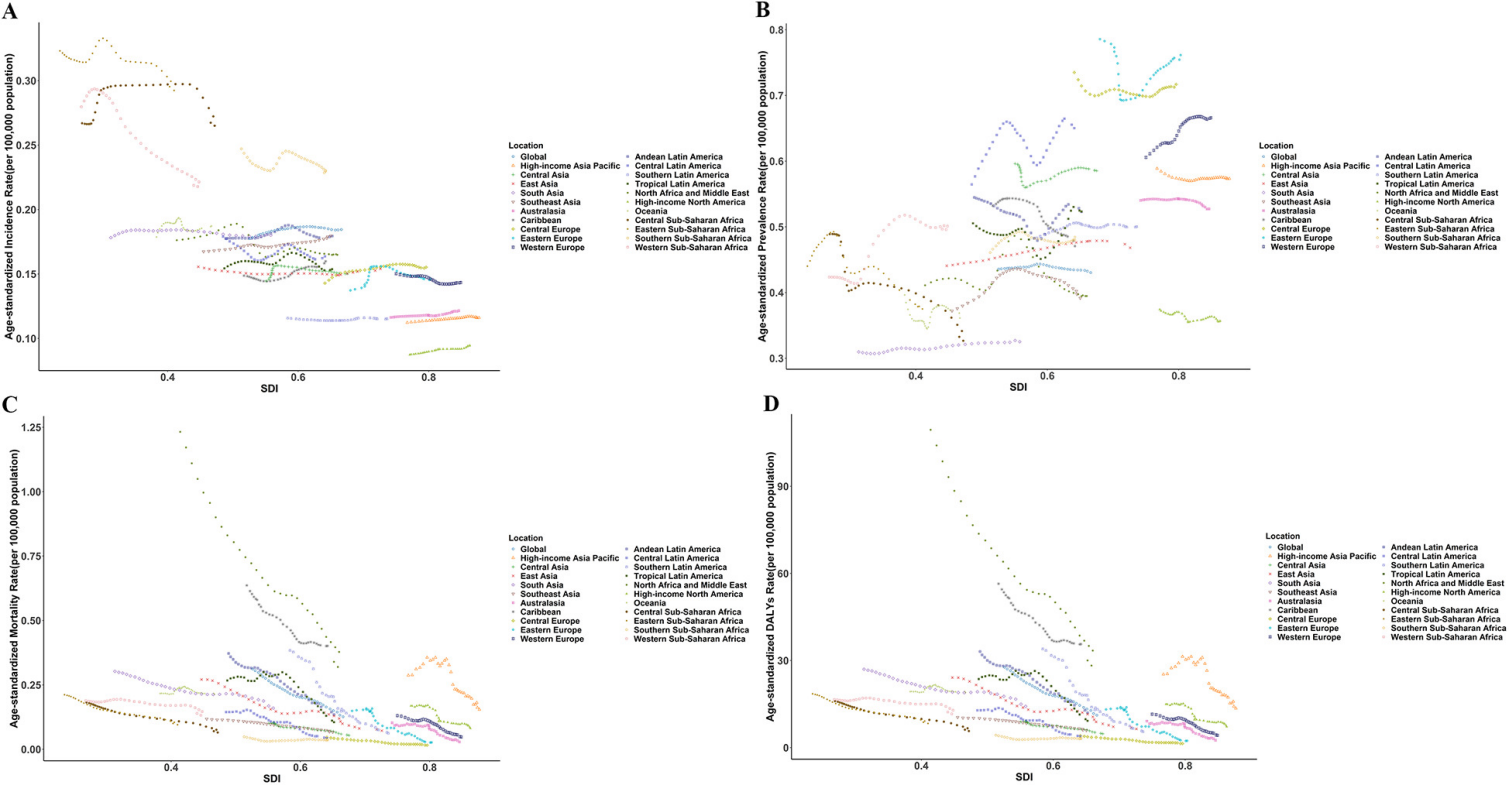
The relationship of age-standardized rates of incidence **(A)**, prevalence **(B)**, mortality **(C)**, and disability-adjusted life years **(D)** of pulmonary artery hypertension in global and 21regions from 1990 to 2021.

At the national level, from 1990 to 2021, Slovakia had the highest increase in ASIR of pediatric PAH, with an average annual trend of 0.77%, followed by Serbia (AAPC 0.71%). Over the same period, Senegal showed the most substantial decrease in ASIR (AAPC −1.74%), followed by Liberia (AAPC −1.44%). Among 204 countries, Switzerland exhibited the highest ASPR of pediatric PAH globally, with values of 1.31 per 100,000 in 1990 and 1.38 per 100,000 in 2021. Iran (Islamic Republic of Iran) experienced the fastest decline in both age-standardized mortality rate (ASMR) and age-standardized disability rate (ASDR), with annual average percentage changes (AAPC) of −7.71% and −7.80%, respectively. Conversely, Tokelau recorded the highest increase in these rates, with AAPCs of 5.42% for ASMR and 5.37% for ASDR (Figure 4).

## Discussion

Globally, there is a scarcity of epidemiological data on pediatric PAH. To our knowledge, this study is the first to thoroughly assess the disease burden of pediatric PAH across 204 countries and territories, examining incidence, prevalence, mortality, and DALYs from 1990 to 2021. Pediatric PAH shares common features of adult disease but is associated with several additional disorders and challenges (4). Therefore, a comprehensive understanding of the globally epidemiology and characteristics of pediatric PAH is essential.

### Global insights on the burden of pediatric PAH

Current epidemiological data on pediatric PAH are mainly stemmed from registry cohorts; thus, research had predominantly focused on specific subtypes, including idiopathic PAH, heritable PAH, drug- and toxin-induced PAH and associated PAH. A report from the UK National Pediatric Pulmonary Hypertension Service highlighted a 20-year experience with pediatric PAH, indicating an incidence rate of 1.56 per million and a prevalence rate of 9.83 per million among individuals aged 0–18 years (17). Utilizing the MarketScan claims database revealed an incidence rate of 4.8–8.1 per million and a prevalence rate of 25.7–32.6 per million in the same age group (12). Alain Fraisse et al. stated that the prevalence of pediatric PAH in France was 3.7 cases per million (8). These values were significantly higher than the incidence and prevalence rates of pediatric PAH observed in our global study. This discrepancy might be attributed to the fact that the aforementioned study data were derived from developed countries, lacking information from other regions globally, which introduced a substantial bias. Our study represented the inaugural report of global epidemiological data on pediatric PAH for individuals aged 0–14 years, thereby addressing a significant gap in the current literature on pediatric PAH epidemiology.

### Age differences in the burden of pediatric PAH

Research from insured pediatric population in the USA indicated that the incidence and prevalence of pediatric PAH were higher in children under 2 years of age, with a significant decline observed after this age (12). A 2022 national registry study from the UK similarly reported age-related variations in incidence, highlighting that the annual incidence rate of PAH in children under 1 year was notably higher than in other age groups, at 5.89 per million (95% CI: 1.08–1.95 per million) (17). Based on the analysis of GBD2021 for pediatric PAH in children aged 0–14 years, we found that the ASIR remained consistent across the following age groups: 0–6 days, 7–27 days, 1–5 months, 6–11 months, 12–23 months, 2–4 years, 5–9 years, and 10–14 years. In contrast, ASPR showed a gradual increase with advancing age and 10–14 years group had the highest prevalence rate. Previous studies had reported significant variability in the age of diagnosis for children with PAH across different registries, with ages ranging from 1.5 to 14.8 years (16, 22, 23). Since children with PAH often cannot actively report discomfort, it was essential for caregivers to observe any abnormal behaviors during routine care. The early, atypical symptoms of PAH can easily be overlooked by parents, leading to delays in diagnosis. The REVEAL study reported a median delay of 9 months from the onset of symptoms to diagnosis in pediatric PAH (22), while the median diagnostic delay for PAH in Chinese children was 24 months (23). The age characteristics of pediatric PAH globally did not align with those observed in specific countries, which may be attributed to variations in awareness, diagnostic capabilities, and unequal distribution of healthcare resources in different countries and regions. These factors could contribute to the discrepancies in prevalence rates across age groups.

### Sex differences in the burden of pediatric PAH

Globally, the prevalence of pediatric PAH was higher in female patients compared to the males. In 1990, the female-to-male ratio was 1.25:1, which had increased to 1.31:1 by 2021 (Table 1). This finding was consistent with previous reports. Studies showed that the female-to-male prevalence ratio was reported to be between 0.9 and 1.7:1 (8, 22), which was lower than the ratio observed in adult PAH, ranging from 1.4 to 4.1:1 (24). A UK study found that among children with PAH under the age of 5, the female-to-male ratio showed no significant difference (0.9–1.1:1). However, in the adolescent groups aged 5–11 years (1.4:1) and 12–18 years (1.8:1), the proportion of females was higher than that of males (17). In conclusion, similar to adult PAH, the prevalence of pediatric PAH was higher in females (25). Although female children had a higher prevalence of pediatric PAH, males exhibited a higher proportion of ASMR (0.25 per 100,000 vs. 0.37 per 100,000 in 1990 and 0.11 per 100,000 vs. 0.15 per 100,000 in 2021) and DALYs (22.00 per 100,000 vs. 33.01 per 100,000 in 1990 and 9.39 vs. 12.86 per 100,000 in 2021). This disparity might be attributed to the protective effects of estrogen, which improved right ventricular (RV) function in PAH by modulating apoptosis, enhancing mitochondrial function, and reducing inflammation and oxidative stress, ultimately leading to improved RV performance (26). In terms of genetic susceptibility, female BMPR2 mutation carriers were more than twice as likely to develop PAH compared to male carriers (27); in a large cohort of individuals with BMPR2 mutations, approximately 70% of the affected population were women (28). Moreover, multiple studies had demonstrated that women exhibit a favorable differential response to PAH-specific therapies, including endothelin receptor antagonists (29), prostacyclin analogues (30), and the phosphodiesterase type 5 inhibitors (31). Additionally, certain systemic diseases, such as connective tissue diseases (CTDs), were associated with the development of PAH. Notably, CTDs were more prevalent among female patients.

### Sociodemographic differences in the burden of pediatric PAH

Globally, countries or regions with high SDI exhibited the lowest incidence, higher prevalence, and lowest DALYs for pediatric PAH. High-income Eastern Europe had the highest prevalence, likely due to advanced healthcare systems that offer superior diagnostic tools, specialized healthcare professionals, and comprehensive treatment options, resulting in more reported cases of pediatric PAH. Additionally, many contemporary PAH registries are situated in economically developed areas of Europe and the US (32, 33). In contrast, low SDI regions, such as Sub-Saharan Africa, experienced the highest PAH incidence, mainly due to limited medical resources, inadequate early diagnosis, and insufficient intervention measures. These factors contribute to a higher number of cases. North Africa and the Middle East showed the highest global PAH-related mortality and DALYs. The diagnosis and treatment of pediatric PAH in this region were significantly impacted by limited medical resources and restricted access to advanced medical interventions (34).The substantial burden of cardiovascular disease further exacerbates PAH risk (35). Moreover, prolonged wars and political instability in many North African and Middle Eastern countries have led to significant infrastructure damage and the collapse of public health systems, severely impacting disease prevention and treatment. Globally, health inequality in PAH exists, and low-SDI countries and regions require more medical resource allocation to address these disparities effectively.

While the GBD project provides epidemiological estimates for pediatric PAH, its limitations warrant consideration. Firstly, the study relies on heterogeneous data inputs, which introduces inherent limitations. Data coverage and quality vary significantly across regions, particularly in low-income countries where disease surveillance systems are underdeveloped. While GBD employs modeling techniques to address gaps, estimates for these regions may still be biased due to reliance on extrapolation primarily. Although GBD provides uncertainty intervals, these may not fully capture structural biases arising from coarse spatial resolution or unmeasured local confounders. Thus, interpretations at finer scales require supplementary local data validation. Secondly, the GBD database lacks racial demographics data, preventing us from assessing racial disparities in the disease burden of PAH.

## Conclusions

Globally, with the exception of incidence, the prevalence, mortality, and DALYs of pediatric PAH all showed a declining trend. Females exhibited a notably higher incidence and prevalence of PAH compared to males with lower mortality and DALYs associated with the condition. ASPR showed a gradual increase with advancing age and 10–14 years group had the highest prevalence rate, however, newborns aged 0–6 days had the highest mortality and DALYs rates. High-SDI countries or regions had the higher prevalence and the lowest mortality and DALYs. It is imperative to tailor health management policies in accordance with these demographic and geographic characteristics.

## Data Availability

Publicly available datasets were analyzed in this study. This data can be found here: http://ghdx.healthdata.org/gbd-results-tool.

## References

[1] Humbert M Kovacs G Hoeper MM Badagliacca R Berger RMF Brida M 2022 ESC/ERS Guidelines for the diagnosis and treatment of pulmonary hypertension. Eur Respir J. (2023) 61(1):2200879. 10.1183/13993003.00879-2022 36028254

[2] Abman SH Hansmann G Archer SL Ivy DD Adatia I Chung WK Pediatric pulmonary hypertension: guidelines from the American Heart Association and American Thoracic Society. Circulation. (2015) 132:2037–99. 10.1161/CIR.0000000000000329 26534956

[3] Simonneau G Montani D Celermajer DS Denton CP Gatzoulis MA Krowka M Haemodynamic definitions and updated clinical classification of pulmonary hypertension. Eur Respir J. (2019) 53:1801913. 10.1183/13993003.01913-2018 30545968 PMC6351336

[4] Rosenzweig EB Abman SH Adatia I Beghetti M Bonnet D Haworth S Paediatric pulmonary arterial hypertension: updates on definition, classification, diagnostics and management. Eur Respir J. (2019) 53(1):1801916. 10.1183/13993003.01916-2018 30545978 PMC6351335

[5] Barst RJ Gibbs JSR Ghofrani HA Hoeper MM McLaughlin VV Rubin LJ Updated evidence-based treatment algorithm in pulmonary arterial hypertension. J Am Coll Cardiol. (2009) 54:S78–s84. 10.1016/j.jacc.2009.04.017 19555861 PMC3686287

[6] Humbert M Sitbon O Simonneau G. Treatment of pulmonary arterial hypertension. N Engl J Med. (2004) 351:1425–36. 10.1056/NEJMra040291 15459304

[7] Ghofrani HA Gomberg-Maitland M Zhao L Grimminger F. Mechanisms and treatment of pulmonary arterial hypertension. Nat Rev Cardiol. (2025) 22(2):105–20. 10.1038/s41569-024-01064-4 39112561

[8] Fraisse A Jais X Schleich JM di Filippo S Maragnès P Beghetti M Characteristics and prospective 2-year follow-up of children with pulmonary arterial hypertension in France. Arch Cardiovasc Dis. (2010) 103:66–74. 10.1016/j.acvd.2009.12.001 20226425

[9] Kwiatkowska J Zuk M Migdal A Kusa J Skiba E Zygielo K Children and adolescents with pulmonary arterial hypertension: baseline and follow-up data from the Polish registry of pulmonary hypertension (BNP-PL). J Clin Med. (2020) 9(6):1717. 10.3390/jcm9061717 32503164 PMC7356296

[10] del Cerro Marín MJ Sabaté Rotés A Rodriguez Ogando A Mendoza Soto A Quero Jiménez M Gavilán Camacho JL Assessing pulmonary hypertensive vascular disease in childhood. Data from the Spanish registry. Am J Respir Crit Care Med. (2014) 190:1421–9. 10.1164/rccm.201406-1052OC 25379685

[11] van Loon RL Roofthooft MT Hillege HL ten Harkel AD van Osch-Gevers M Delhaas T Pediatric pulmonary hypertension in The Netherlands: epidemiology and characterization during the period 1991 to 2005. Circulation. (2011) 124:1755–64. 10.1161/CIRCULATIONAHA.110.969584 21947294

[12] Li L Jick S Breitenstein S Hernandez G Michel A Vizcaya D. Pulmonary arterial hypertension in the USA: an epidemiological study in a large insured pediatric population. Pulm Circ. (2017) 7:126–36. 10.1086/690007 28680572 PMC5448526

[13] Wijeratne DT Lajkosz K Brogly SB Lougheed MD Jiang L Housin A Increasing incidence and prevalence of World Health Organization groups 1 to 4 pulmonary hypertension: a population-based cohort study in Ontario, Canada. Circ Cardiovasc Qual Outcomes. (2018) 11:e003973. 10.1161/CIRCOUTCOMES.117.003973 29444925 PMC5819352

[14] Pektas A Pektas BM Kula S. An epidemiological study of paediatric pulmonary hypertension in Turkey. Cardiol Young. (2016) 26:693–7. 10.1017/S1047951115001043 26088722

[15] Berger RM Beghetti M Humpl T Raskob GE Ivy DD Jing ZC Clinical features of paediatric pulmonary hypertension: a registry study. Lancet. (2012) 379:537–46. 10.1016/S0140-6736(11)61621-8 22240409 PMC3426911

[16] Abman SH Mullen MP Sleeper LA Austin ED Rosenzweig EB Kinsella JP Characterisation of paediatric pulmonary hypertensive vascular disease from the PPHNet registry. Eur Respir J. (2021) 59(1):2003337. 10.1183/13993003.03337-2020 34140292 PMC10335325

[17] Constantine A Dimopoulos K Haworth SG Muthurangu V Moledina S. Twenty-Year experience and outcomes in a national pediatric pulmonary hypertension service. Am J Respir Crit Care Med. (2022) 206:758–66. 10.1164/rccm.202110-2428OC 35579610 PMC9799107

[18] GBD 2021 Diseases and Injuries Collaborators. Global incidence, prevalence, years lived with disability (YLDs), disability-adjusted life-years (DALYs), and healthy life expectancy (HALE) for 371 diseases and injuries in 204 countries and territories and 811 subnational locations, 1990–2021: a systematic analysis for the global burden of disease study 2021. Lancet. (2024) 403:2133–61. 10.1016/S0140-6736(24)00757-8 38642570 PMC11122111

[19] GBD 2021 Risk Factors Collaborators. Global burden and strength of evidence for 88 risk factors in 204 countries and 811 subnational locations, 1990–2021: a systematic analysis for the global burden of disease study 2021. Lancet. (2024) 403:2162–203. 10.1016/S0140-6736(24)00933-4 38762324 PMC11120204

[20] Kim HJ Fay MP Feuer EJ Midthune DN. Permutation tests for joinpoint regression with applications to cancer rates. Stat Med. (2000) 19:335–51. 10.1002/(sici)1097-0258(20000215)19:3<335::aid-sim336>3.0.co;2-z 10649300

[21] Xie J Wang M Long Z Ning H Li J Cao Y Global burden of type 2 diabetes in adolescents and young adults, 1990–2019: systematic analysis of the global burden of disease study 2019. BMJ. (2022) 379:e072385. 10.1136/bmj-2022-072385 36740855 PMC9727920

[22] Barst RJ McGoon MD Elliott CG Foreman AJ Miller DP Ivy DD. Survival in childhood pulmonary arterial hypertension: insights from the registry to evaluate early and long-term pulmonary arterial hypertension disease management. Circulation. (2012) 125:113–22. 10.1161/CIRCULATIONAHA.111.026591 22086881

[23] Qian Y Quan R Chen X Gu Q Xiong C Han H Characteristics, long-term survival, and risk assessment of pediatric pulmonary arterial hypertension in China: insights from a national multicenter prospective registry. Chest. (2023) 163:1531–42. 10.1016/j.chest.2022.11.038 36470418

[24] Hester J Ventetuolo C Lahm T. Sex, gender, and sex hormones in pulmonary hypertension and right ventricular failure. Compr Physiol. (2019) 10:125–70. 10.1002/cphy.c190011 31853950 PMC7338988

[25] GBD 2021 Pulmonary Arterial Hypertension Collaborators. Global, regional, and national burden of pulmonary arterial hypertension, 1990–2021: a systematic analysis for the global burden of disease study 2021. Lancet Respir Med. (2025) 13(1):69–79. 10.1016/S2213-2600(24)00295-9 39433052 PMC11698691

[26] Keen J Prisco SZ Prins KW. Sex differences in right ventricular dysfunction: insights from the bench to bedside. Front Physiol. (2020) 11:623129. 10.3389/fphys.2020.623129 33536939 PMC7848185

[27] Best DH Sumner KL Smith BP Damjanovich-Colmenares K Nakayama I Brown LM *EIF2AK4* mutations in patients diagnosed with pulmonary arterial hypertension. Chest. (2017) 151:821–8. 10.1016/j.chest.2016.11.014 27884767

[28] Evans JD Girerd B Montani D Wang XJ Galiè N Austin ED *BMPR2* mutations and survival in pulmonary arterial hypertension: an individual participant data meta-analysis. Lancet Respir Med. (2016) 4:129–37. 10.1016/S2213-2600(15)00544-5 26795434 PMC4737700

[29] Gabler NB French B Strom BL Liu Z Palevsky HI Taichman DB Race and sex differences in response to endothelin receptor antagonists for pulmonary arterial hypertension. Chest. (2012) 141:20–6. 10.1378/chest.11-0404 21940766 PMC5991545

[30] Frantz RP Schilz RJ Chakinala MM Badesch DB Frost AE McLaughlin VV Hospitalization and survival in patients using epoprostenol for injection in the PROSPECT observational study. Chest. (2015) 147:484–94. 10.1378/chest.14-1004 25320967 PMC4314821

[31] Mathai SC Hassoun PM Puhan MA Zhou Y Wise RA. Sex differences in response to tadalafil in pulmonary arterial hypertension. Chest. (2015) 147:188–97. 10.1378/chest.14-0263 25122150 PMC4285077

[32] Frost AE Badesch DB Barst RJ Benza RL Elliott CG Farber HW The changing picture of patients with pulmonary arterial hypertension in the United States: how REVEAL differs from historic and non-US contemporary registries. Chest. (2011) 139:128–37. 10.1378/chest.10-0075 20558556

[33] Hoeper MM Huscher D Pittrow D. Incidence and prevalence of pulmonary arterial hypertension in Germany. Int J Cardiol. (2016) 203:612–3. 10.1016/j.ijcard.2015.11.001 26580339

[34] Hoeper MM Humbert M Souza R Idrees M Kawut SM Sliwa-Hahnle K A global view of pulmonary hypertension. Lancet Respir Med. (2016) 4:306–22. 10.1016/S2213-2600(15)00543-3 26975810

[35] Abboud M Karam S. Hypertension in the Middle East: current state, human factors, and barriers to control. J Hum Hypertens. (2022) 36:428–36. 10.1038/s41371-021-00554-z 34075186

