# Correction: Pediatric pulmonary arterial hypertension: global epidemiology and disease burden during the period 1990 to 2021

**DOI:** 10.3389/fcvm.2025.1703514

**Published:** 2025-10-08

**Authors:** Ying Chen, Rongguo Zhang, Yongxiao Zheng, Chenghong Li, Xiaobei Wang, Zhongmei Wen, Fajiu Li

**Affiliations:** ^1^ Department of Pulmonary and Critical Care Medicine, The Sixth Hospital of Wuhan, Affiliated Hospital of Jianghan University, Wuhan, Hubei, China; ^2^ Department of Biostatistics, School of Public Health, Cheeloo College of Medicine, Shandong University, Jinan, Shandong, China; ^3^ Institute for Medical Dataology, Cheeloo College of Medicine, Shandong University, Jinan, Shandong, China; ^4^ Department of Respiratory Medicine, The First Hospital of Jilin University, Changchun, Jilin, China

**Keywords:** pulmonary arterial hypertension, epidemiology, pediatric, global, disease burden

A Correction on Pediatric pulmonary arterial hypertension: global epidemiology and disease burden during the period 1990 to 2021 By Chen Y, Zhang R, Zheng Y, Li C, Wang X, Wen Z and Li F (2025). Front. Cardiovasc. Med. 12:1544545. doi: 10.3389/fcvm.2025.1544545

An incorrect number was provided for the Natural Science Foundation of Wuhan. The correct number is 2023020201020561.

The original version of this article has been updated.

