# Supplementary material for: Pediatric pulmonary arterial hypertension: global epidemiology and disease burden during the period 1990 to 2021
Source: Front Cardiovasc Med. 2025 Aug 29;12:1544545. doi: 10.3389/fcvm.2025.1544545 (PMC12427251; doi:10.3389/fcvm.2025.1544545)
Supplement: Supplementary file 1 [file Table1.docx]

Supplementary Table S1 Global cases and crude rates of pulmonary artery hypertension in incidence, prevalence, mortality and DALYs.

|  | **Incidence (95%UI)** | | | |
| --- | --- | --- | --- | --- |
|  | **No of pediatric PAH in 1990** | **Rate in 1990 (per 100,000)** | **No of pediatric PAH in 2021** | **Rate in 2021 (per 100,000)** |
| **Incidence** | | | | |
| 0-6 days | 4.51(6.58,2.87) | 0.18(0.26,0.11) | 4.49(6.55,2.88) | 0.18(0.27,0.12) |
| 7-27 days | 13.31(19.41,8.5) | 0.18(0.26,0.11) | 13.36(19.45,8.6) | 0.18(0.27,0.12) |
| 1-5 months | 96.81(140.18,63.33) | 0.18(0.26,0.12) | 98.45(142.42,64.84) | 0.18(0.26,0.12) |
| 6-11 months | 111.86(159.89,75.84) | 0.18(0.25,0.12) | 115.74(165.37,78.8) | 0.18(0.26,0.12) |
| 12-23 months | 220.45(306.28,155.98) | 0.18(0.25,0.13) | 235.18(326.93,166.83) | 0.18(0.25,0.13) |
| 2-4 years | 649.84(853.49,479.53) | 0.18(0.23,0.13) | 737(968.5,543.42) | 0.18(0.24,0.13) |
| 5-9 years | 1036.22(1375.5,750.02) | 0.18(0.24,0.13) | 1262.72(1677.33,914.05) | 0.18(0.24,0.13) |
| 10-14 years | 954.75(1476.42,565.68) | 0.18(0.28,0.11) | 1241.34(1927.97,735.17) | 0.19(0.29,0.11) |
| **Prevalence** | | | | |
| 0-6 days | 0.04(0.06,0.03) | 0(0,0) | 0.04(0.06,0.03) | 0(0,0) |
| 7-27 days | 0.62(0.9,0.4) | 0.01(0.01,0.01) | 0.62(0.9,0.4) | 0.01(0.01,0.01) |
| 1-5 months | 24.65(35.21,15.99) | 0.05(0.06,0.03) | 24.89(35.47,16.25) | 0.05(0.07,0.03) |
| 6-11 months | 65.87(93.48,43.88) | 0.1(0.15,0.07) | 67.28(95.44,45.08) | 0.11(0.15,0.07) |
| 12-23 months | 218.39(303.99,149.2) | 0.18(0.24,0.12) | 227.77(316.66,154.93) | 0.18(0.25,0.12) |
| 2-4 years | 1094.18(1483.67,781.43) | 0.3(0.4,0.21) | 1202.09(1646.66,858.03) | 0.3(0.41,0.21) |
| 5-9 years | 2675.03(3507.3,1930.34) | 0.46(0.6,0.33) | 3133.23(4120.2,2248.5) | 0.46(0.6,0.33) |
| 10-14 years | 3535.88(4992.66,2364.52) | 0.66(0.93,0.44) | 4317.65(6154.21,2876.12) | 0.65(0.92,0.43) |
| **Mortality** | | | | |
| 0-6 days | 867.33(1093.63,653.37) | 34.16(43.07,25.73) | 335.06(454.63,252.04) | 13.67(18.55,10.28) |
| 7-27 days | 381.49(518.79,212.8) | 5.09(6.92,2.84) | 138.99(173.35,108.22) | 1.91(2.38,1.48) |
| 1-5 months | 966.81(1377.58,531.89) | 1.77(2.52,0.97) | 366.78(452.07,288.56) | 0.68(0.84,0.54) |
| 6-11 months | 682.32(1151.19,315.7) | 1.08(1.82,0.5) | 243.3(344.19,156.01) | 0.39(0.54,0.25) |
| 12-23 months | 398.37(630.86,228.18) | 0.32(0.51,0.18) | 170.38(219.48,129.94) | 0.13(0.17,0.1) |
| 2-4 years | 319.78(695.27,148.34) | 0.09(0.19,0.04) | 154.93(274.12,83.22) | 0.04(0.07,0.02) |
| 5-9 years | 245.47(323.22,176.05) | 0.04(0.06,0.03) | 151.2(188.25,121.5) | 0.02(0.03,0.02) |
| 10-14 years | 187.75(236.07,141.41) | 0.04(0.04,0.03) | 153.92(193.77,121.75) | 0.02(0.03,0.02) |
| **DALYs** | | | | |
| 0-6 days | 78038.87(98399.52,58787.04) | 3073.56(3875.46,2315.32) | 30147.01(40905.31,22677.85) | 1229.88(1668.78,925.17) |
| 7-27 days | 34324.14(46677.03,19146.5) | 457.62(622.32,255.27) | 12505(15596.55,9737.32) | 171.44(213.83,133.5) |
| 1-5 months | 86784.02(123654.85,47745.63) | 158.96(226.49,87.45) | 32925(40580.18,25903.79) | 61.25(75.49,48.19) |
| 6-11 months | 60950.21(102829.88,28204.72) | 96.57(162.93,44.69) | 21737.88(30752.16,13941.56) | 34.4(48.66,22.06) |
| 12-23 months | 35308.28(55903.34,20231.69) | 28.33(44.86,16.23) | 15113.89(19462.91,11528.64) | 11.77(15.16,8.98) |
| 2-4 years | 27861.94(60507.67,12977.3) | 7.58(16.46,3.53) | 13527.95(23842.3,7335.98) | 3.36(5.92,1.82) |
| 5-9 years | 20588.43(27100.65,14825.83) | 3.53(4.64,2.54) | 12812.78(15867.34,10347.53) | 1.86(2.31,1.51) |
| 10-14 years | 14885.35(18570.03,11379.87) | 2.78(3.47,2.12) | 12328.61(15345.22,9823.25) | 1.85(2.3,1.47) |

No= number
